# Clinical values of different specimen preparation methods for the diagnosis of lung cancer by EBUS-TBNA

**DOI:** 10.1186/s13000-024-01486-1

**Published:** 2024-04-19

**Authors:** Hansheng Wang, Jiankun Wang, Yan Liu, Yunyun Wang, Yanhui Zhou, Dan Yu, Hui You, Tao Ren, Yijun Tang, Meifang Wang

**Affiliations:** 1 Department of Pulmonary and Critical Care Medicine, Taihe Hospital, Hubei University of Medicine, Shiyan, 442000 Hubei P.R. China; 2 Department of Thoracic surgery, Taihe Hospital, Hubei University of Medicine, Shiyan, 442000 Hubei P.R. China; 3 Department of Pathology, Taihe Hospital, Hubei University of Medicine, Shiyan, 442000 Hubei P.R. China

**Keywords:** hilar/mediastinal lymphadenopathy, Endobronchial ultrasound-guided transbronchial needle aspiration, Lung cancer, Traditional smears cytology, Liquid-based cytology

## Abstract

**Background and objective:**

EBUS-TBNA has emerged as an important minimally invasive procedure for the diagnosis and staging of lung cancer. Our objective was to evaluate the effect of different specimen preparation from aspirates on the diagnosis of lung cancer.

**Methods:**

181 consecutive patients with known or suspected lung cancer accompanied by hilar / mediastinal lymphadenopathy underwent EBUS-TBNA from January 2019 to December 2022. Specimens obtained by EBUS-TBNA were processed by three methods: Traditional smear cytology of aspirates (TSC), liquid-based cytology of aspirates (LBC) and histopathology of core biopsies.

**Results:**

EBUS-TBNA was performed in 181 patients on 213 lymph nodes, the total positive rate of the combination of three specimen preparation methods was 80.7%. The diagnostic positive rate of histopathology was 72.3%, TSC was 68.1%, and LBC was 65.3%, no significant differences was observed (*p* = 0.29); however, statistically significant difference was noted between the combination of three preparation methods and any single specimen preparation methods (*p* = 0.002). The diagnostic sensitivity of histopathology combined with TSC and histopathology combined with LBC were 96.5 and 94.8%, the specificity was 95.0% and 97.5%, the PPV was 98.8% and 99.4%, the NPV was 86.4% and 81.2%, the diagnostic accuracy was 96.2% and 95.3%, respectively; The sensitivity and accuracy of above methods were higher than that of single specimen preparation, but lower than that of combination of three preparation methods.

**Conclusion:**

When EBUS-TBNA is used for the diagnosis and staging of lung cancer, histopathology combined with TSC can achieve enough diagnostic efficiency and better cost-effectiveness.

**Supplementary Information:**

The online version contains supplementary material available at 10.1186/s13000-024-01486-1.

## Introduction

Endobronchial ultrasound guided transbronchial needle aspiration (EBUS-TBNA) is a minimally invasive and well established procedure for the diagnosis and staging of lung cancer, which has been clinically developed and has achieved positive clinical effects [1, 2]. Histopathology, traditional smear cytology and liquid-based cytology (LBC) of aspirates are the main methods for clinical application. However, TSC interpretation is at times limited by the presence of air-drying artefacts, mucous, blood, and cellular overlap [3]. LBC, as an extensively used cytopathologic technique, is initially introduced for screening cervical cancer [4], and has been increasingly used for exfoliative and non-gynecologic cytology in recent decades due to clean background of smear and well-preserved nuclear details, which got more reliable and feasible results compared with conventional smears [5]. Nevertheless, some studies indicated that LBC did not perform better than TSC in terms of diagnostic efficiency [6], while others suggested similar results [7, 8]; others favor the high diagnostic efficiency of LBC [3]. It has also been shown that the combination of EBUS-TBNA cytology and histopathology can significantly improve the diagnostic accuracy of lung cancer. However, the diagnostic effectiveness and cost-effectiveness of the combination of the three methods are unknown due to limited research data [9]. In addition to histopathology, whether cytological specimen preparation methods are required and whether two different cytological specimen preparation methods are both necessary; And how different preparation methods of specimens affect diagnosis, effectiveness, and consistency of results are also unknown. Herein, we manage EBUS-TBNA material with three specimen preparation methods and analyze their diagnostic efficiency and consistency for lung cancer, and look forward to provide some reference for future clinical practice.

## Materials and methods

### Study population and EBUS-TBNA procedure

**Fig. 1:**
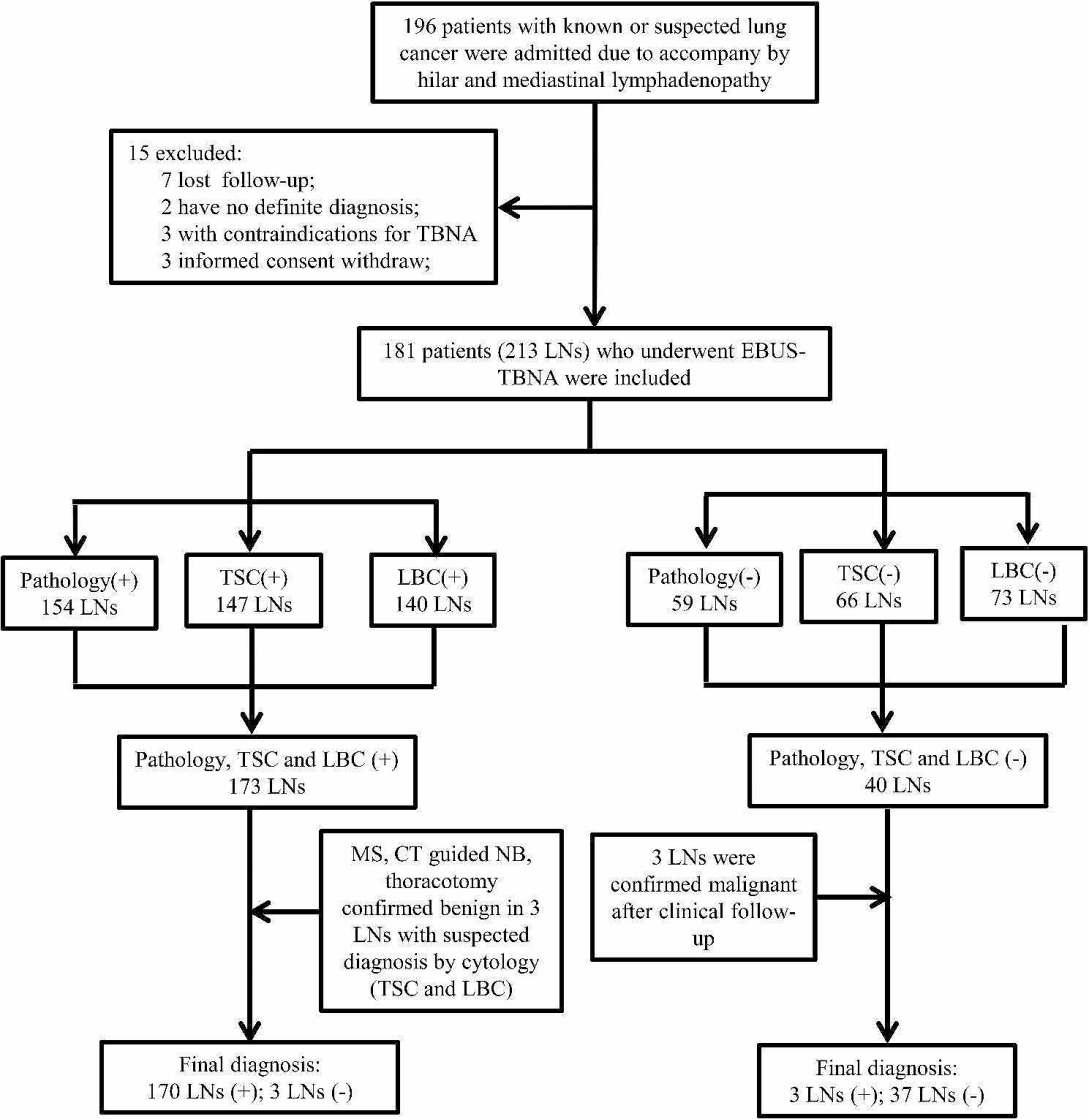
Study flowchart and diagnostic process

We retrospectively analyzed the records of a total of 196 patients with suspected hilar or/and mediastinal lymph node involvement who underwent EBUS-TBNA for diagnosis and staging of lung cancer from January 2019 to December 2022 at the Department of Pulmonary and Critical Care Medicine, Taihe hospital. All patients were evaluated by contrast-enhanced computed tomography (CT) scan of the chest and upper abdomen, bone radioisotope scanning, brain magnetic resonance imaging (MRI), and routine flexible fiberoptic bronchoscopy. Subsequently, EBUS-TBNA was performed in patients with radiologically defined enlargement of mediastinal and/or hilar lymph nodes with a short axis of 5 mm or more on contrast-enhanced chest CT [10]. Clinical staging of lung cancer according to the International TNM staging system reported by Mountain and Dressler [11]. Exclusion criteria: patients with contraindications to bronchoscopy [12, 13], no written informed consent obtained from patients, absence of further confirmatory surgical procedures after an inconclusive TBNA result, and lost follow-up. Finally, 181 patients were included, study flowchart and diagnostic process is shown in Fig. 1. The retrospective study was approved by the Ethics Committee of Taihe Hospital.

Before bronchoscopy procedures, local anesthesia is achieved by nebulizing inhalation of 2% lidocaine solution plus 4% lidocaine solution sprayed into the pharynx of patients, and conscious sedation with intravenous midazolam. Standard flexible bronchoscopy was performed to complete the inspection of airway first, and secretions were thoroughly cleared to reduce interference with subsequent ultrasound bronchoscopy. Inflating the balloon (MAJ-1351; Olympus, Tokyo, Japan) attached to EBUS probe of bronchoscope (BF-UC260FW; Olympus, Tokyo, Japan) with saline solution first, the ultrasound bronchoscope was then orally inserted, the balloon was brought into contact with the airway wall and moved in all directions to identify the lesions for sampling. After identifying the target lymph node by EBUS, the size of the lesion and the puncture distance were measured, color Doppler is used to observe blood flow in and around the lesion area, absolutely avoiding blood vessels. A 22-gauge needle (NA-201SX- 4022, Olympus) was passed through the instrument channel of the endoscope, which was then advanced through the tracheobronchial wall into the target lesion under real-time EBUS visualization. After needle piercing the lymph node, the internal stylet is removed and negative pressure is applied with the syringe, and the needle is then moved back and forth within the lesion for sampling. 3 to 5 passes were performed per lesion as suggested by literature [14, 15] for standard EBUS-TBNA procedure. After sampling, the syringe is detached and the needle is retrieved. Nodal location was recorded, the location of the lymph nodes according to the American Thoracic Society mediastinal map [11]. All procedures were performed by or under the supervision of the same pulmonologist. The vital signs of each patient were monitored during the procedure, including blood pressure, heart rate, respiratory rate, and oxygen saturation.

### Specimens’ management

#### Traditional direct smear method production cytology

The tissue obtained by the every needle punctures was pushed onto the glass-slide with the needle core, the aspirated material was smeared onto sterile glass-slide by the direct smear method and air-dried as well as fixed in 95% ethanol for 15 min, stained with hematoxylin-eosin (H&E) and observed under a light microscope (CX31, Olympus Corporation). Two cytology specimens were prepared per site.

#### Liquid thin-layer cytology technology production

The tissue obtained by the every needle punctures was pushed onto the glass-slide with the needle core, the residual aspirates (mainly fragments) stored at the lumen of the needle and catheter was then washed with physiological saline and transferred into the liquid base testing bottle (Thinprep preservation solution) for cytological analysis with method of LBC, which is prepared by TCT microcomputer processing system. The detailed process is as follows: the sample was centrifuged at a radius of 10 cm at 1500 r/min for 5 min and the supernatant was discarded; 25 ml of cleaning fluid was added and then oscillated prior to centrifugation at 1500 r/min for 5 min; the supernatant was discarded again, and the sediment was transferred into a Thinprep liquid then oscillated and mixed. After 15 min, an ultrathin cell smear was made by a TCT microcomputer processing system, fixed with 95% ethanol for 15 min, stained with Papanicolaou (Pap) stain, sealed, and observed under a light microscope (CX31, Olympus Corporation). If necessary, cell blocks were made.

#### Histopathological examination

Then all remaining aspirates and tissue cores were collected and transferred into tissue preservation containers filled with 10% formalin, embedded with paraffin, 3 μm-thick continuous sectioning, H&E staining, microscopic examination, and immunohistochemical examination when necessary.

### Diagnostic criteria

Histopathological (histopathology) and cytological (TSC and LBC) slide preparations were reviewed by two senior pathologists, who were blinded to the patient details and discussed any discrepancies to reach a consensus diagnosis. The final TBNA diagnosis was based on the analysis of the combination of histopathology, TSC and LBC. Classification of lung cancer was based on morphological appearances (H&E stain), and immunohistochemistry was performed when necessary. The diagnostic categories were as follows: positive (definite malignant tumor cells were detected), suspicious (reported as suspected cancer cells), negative (lymphoid cells or inflammatory cells or anthracotic pigment-laden macrophages or granulomatous inflammation were detected, no tumor cells were reported), non-diagnostic (a lot of respiratory tract mucosal cells and/or chondromyxoid fragments of cartilage presenting in a single field or presented as erythrocytes only in whole field). In our study, EBUS-TBNA results were considered positive when definite malignant tumor cells were detected by cell or histopathological examination. Pathologic findings of highly suspicious malignant cells and clinical manifestations of highly suspected lung cancer or other histologic or cytologic examination proving lung cancer were also considered positive for EBUS-TBNA results and final diagnosis. If TBNA failed to conclude a definite diagnosis (nondiagnostic, or negative results), or produced a non-specific diagnosis of malignancy, patients were referred to CT-guided needle biopsy or surgical procedures (e.g., thoracoscopy, thoracotomy, mediastinoscopy). Surgical histology was regarded as the gold standard. If no definite diagnosis was found after all examinations, at least 6 months of clinical follow-up was required. We categorized the histological and cytological subtypes in accordance with the International association for the study of lung cancer/American thoracic society/European respiratory society (IASLC/ATS/ ERS) [16].

### Statistical analysis

SPSS software version 26.0 was used for statistical analysis. Measurement data are expressed as the mean ± standard deviation (mean ± SD); the chi-square test or Fisher’s exact test was used to compare categorical data. Receiver operator characteristic (ROC) curves were designed to assess sensitivity, specificity, positive predictive values (PPV) and negative predictive values (NPV) for the estimated parameters. A chi-square test was used to compare diagnostic accuracy rates between the different specimen preparation methods. Consistency between the diagnosis of different specimen preparation methods and final diagnosis was assessed by calculating a κ-score. Probability values < 5% (*p* < 0.05) were considered statistically significant.

## Results

### Parameters of patients and lesions

In the present study, there were 196 patients, of whom 15 were excluded, a total of 181 patients (213 lymph nodes) were eventually included, as displayed in Fig. 1. There were 129 males and 52 females, with a median age of 62.1 ± 9.8 years (range, 26–80 years), the clinical characteristics of the included patients are shown in Table 1. Among the 181 patients (213 lymph nodes), there were 28 patients with two stations of lymph nodes puncture samples, 2 patients with three stations of lymph nodes puncture samples, and the remaining 151 patients with one station of lymph node puncture samples, accounting for 15.5%, 1.1% and 83.4% respectively. According to the anatomic site, trachea mediastinal lymph nodes (2 L, R and 4 L, R) accounted for 39.35%, 7 group lymph nodes accounted for 23.9%, hilar lymph nodes (10 L, R and 11 L, R and 12 L, R) accounted for 24.4%, and masses accounted for 12.2%, characteristics of lymph nodes were summarized in Table 1. According to the final diagnosis, there were 173 malignant lymph nodes and 40 benign lymph nodes, the details are as follows: 155 (72.8%) metastatic lung cancer, 5 (2.3%) metastatic extrathoracic cancer, 6 (2.8%) lymphomas, 40 (18.8%) benign and 7 (3.3%) cancer type that unable divided, the detailed classification of cancer cell types is shown in Table 1. The detailed information of tumor subtype that diagnosed by the 3 different tests is shown in Supplemental tables.

**Table 1:** Characteristics of patient population and LNs in included patients (n _patients_=181, n _LNs_ =213)

Baseline characteristics			
Patients (male/female)		181 (129/52)	
Mean age (range), years		61.5 ± 9.9 (26–79)	
Smoking history			
Never smoker		65(35.9%)	
Ex-smoker		85(47.0%)	
Current smoker		31(17.1%)	
Known/suspected lung cancer		59/122	
Station of LNs			
2 L		1(0.45%)	
2R		5(2.3%)	
4 L		13(6.1%)	
4R		65(30.5%)	
7		51(23.9%)	
10 L		2(0.9%)	
10R		13(6.1%)	
11 L		8 (3.8%)	
11R		10(4.7%)	
12 L		6(2.8%)	
12R		13(6.1%)	
Mass		26(12.2%)	
Number of LNs station per patient			
1 station		151(93.1%)	
2 stations	28(6.3%)		
3 stations	2(0.6%)		
Final diagnosis for LNs, n (%)		Metastatic lung cancer 155 (72.8)^**§**^	
		Metastatic extrathoracic cancer 5 (2.3)^**†**^	
		Cancer type unknown 7 (3.3)	
		Lymphoma 6 (2.8)^**❈**^	
		Non-malignancy 40 (18.8)^**‡**^	

Data are presented as n (%) or mean ± SD (range). abbreviations: LNs = Lymph nodes, SqCC = squamous cell carcinoma, AdC = adenocarcinoma, SCLC = small cell lung carcinoma, NSCLC = non-small cell lung carcinoma, LCLC = large cell lung carcinoma

^**§**^including 82 LNs of metastatic lung AdC, 26 LNs of metastatic lung SqCC, 39 LNs of metastatic SCLC, 2 LNs of NSCLC, 2 LNs of metastatic malignant pleural mesothelioma, 1 LN of metastatic lung adeno-squamous carcinoma, 1 LN of metastatic SqCC of the thymus, 1 LN of metastatic LCLC, 1 LN of metastatic lung sarcomatoid carcinoma

^**†**^including 1 LN of metastatic colon adenocarcinoma, 1 LN of metastatic SqCC of the esophagus, 1 LN of metastatic SqCC of the cervix, 1 LN of metastatic gastric adenocarcinoma, 1 LN of metastatic clear cell renal cell carcinoma

^**❈**^ including 5 LNs of B cell non-Hodgkin’s lymphoma, 1 LN of Hodgkin’s lymphoma

^**‡**^including 11 LNs of necrotizing granuloma, 2 LNs of non-necrotizing granuloma, 10 LNs of nonspecific lymphadenopathy, and 17 LNs of anthracotic pigment deposition

### Diagnostic efficiency of different specimen preparation methods

As detailed in Table 2, the positive rate of histopathology, TSC and LBC in the diagnosis of lung cancer with EBUS-TBNA material was 72.3% (154/213), 69.0% (147/213) and 65.7% (140/213), respectively; there was no significant difference (*p* = 0.29) in diagnostic positive rate among the single specimen preparation methods. The positive rate of histopathology combined with TSC or LBC was 78.9% (168/213) and 77.0% (164/213), respectively, the positive rate of combination of the three specimen preparation methods was 81.2%; and no significant difference was noted among them (*p* = 0.56). However, significant difference was observed between combination of the two (histopathology with TSC) or three specimen preparation methods and any single specimen preparation method on diagnostic positive rates (*p* = 0.020 or *p* = 0.003). There were 13 LNs and 3 LNs that histopathology diagnosed as negative but cytology (TSC or LBC) interpreted as definite cancer and suspected cancer, respectively; the 3 LNs that interpretated as suspected cancer by cytology were confirmed as benign after invasive procedure (e.g., CT guided needle biopsy, thoracotomy and mediastinoscopy); there were 40 LNs with negative diagnosis by both histopathology and cytology (TSC or LBC), of them, 3 LNs were confirmed malignant after resected or re-TBNA during a follow-up period ranging from 6 months to 2 years, as shown in Fig. 1. As a result, 213 LNs consisted of 173 malignant LNs and 40 benign LNs. As shown in Table 3; Fig. 1, based on the final diagnosis, 59 negative lymph nodes were reviewed and diagnosed with the original histology slides, of them, 19 were eventually confirmed malignant by VATS resection with lymph node dissection, and a few cases were determined malignant by mediastinoscopy, but no false positive, with a sensitivity of 89.0%, specificity of 100%, PPV of 100%, NPV of 67.8% and diagnostic accuracy of 91.1% for histopathology. Among 147 positive LNs diagnosed by TSC, 2 LNs were false positive, 28 out of 66 negative diagnoses were false negatives, with a sensitivity of 83.8%, specificity of 95.0%, PPV of 98.6%, NPV of 57.6% and diagnostic accuracy of 85.9% for TSC. Among 140 positive results diagnosed by LBC, 1 case was false positive; of the 73 negative diagnoses, 34 were false negatives, with a sensitivity of 80.3%, specificity of 97.5%, PPV of 99.3%, NPV of 53.3% and diagnostic accuracy of 83.6% for LBC. The sensitivity, specificity, PPV, NPV and diagnostic accuracy of histopathology combined with TSC were 97.1%, 95.0%, 98.8%, 86.4% and 96.2%, respectively; the sensitivity, specificity, PPV, NPV and diagnostic accuracy of histopathology combined with LBC were 94.8%, 97.5%, 99.4%, 81.2% and 95.3% respectively; while the sensitivity, specificity, PPV, NPV and diagnostic accuracy of combination of the three specimen preparation methods were 98.3%, 92.5%, 98.3%, 98.3% and 92.5%, respectively. There was no significant difference among histopathology, TSC and LBC on diagnostic accuracy (*p* = 0.064), and there was no significant difference among combination of the three specimen preparation methods and combination of the two specimen preparation methods on diagnostic accuracy (*p* = 0.595); however, significant difference was noted between combination of the two or three specimen preparation methods and any single specimen preparation method on diagnostic accuracy (*p* = 0.000, *p* = 0.000 or *p* = 0.000, respectively), as detailed in Table 3.

**Table 2:** Comparison of the diagnostic positive rates of histopathology, TSC and LBC in the diagnosis of LNs specimen (*n* = 213)

	Positive, n (%)	Negative, n (%)	Diagnostic positive rate, n (%)	χ^2^	*p* value
HP	72.3(154)	27.7(59) ^**a**^	72.3 (154)		
TSC	69.0(147) ^**b**^	31.0(66) ^**c**^	69.0 (147)		
LBC	65.7(140) ^**d**^	34.3(73) ^**e**^	65.7 (140)	2.152^**#**^	0.341^**#**^
HP + TSC	78.9(168) ^**b**^	21.1(45) ^**f**^	78.9 (168)	9.874^**##**^	0.020^**##**^
HP + LBC	77.0(164) ^**d**^	23.0(49) ^**g**^	77.0 (164)	7.178^**###**^	0.066^**###**^
HP + TSC + LBC	81.2(173) ^**h**^	18.8(40) ^**k**^	81.2 (173)	1.152^*^, 14.11^**&**^	0.562^*^,0.003^**&**^
Final diagnosis	81.2(173)	18.8(40)	81.2 (173)		

Abbreviations; HP = Histopathology, TSC = Traditional smear cytology, LBC = Liquid-based cytology

^**a**^ including 19 LNs of false negative diagnosis; ^**b**^ including 2 LNs of false positive diagnosis; ^**c**^ including 28 LNs of false negative diagnosis; ^**d**^ including 1 LN of false positive diagnosis; ^**e**^ including 34 LNs of false negative diagnosis; ^**f**^ including 6 LNs of false negative diagnosis; ^**g**^ including 9 LNs of false negative diagnosis; ^**h**^ including 3 LNs of false positive diagnosis; ^**k**^ including 3 LNs of false negative diagnosis

^**#**^Comparison of diagnostic positive rates among HP, TSC and LBC; ^**##**^Comparison of diagnostic positive rates between combination of the two specimen preparation methods (HP + TSC) and any single specimen preparation method; ^**###**^ Comparison of diagnostic positive rates between combination of the two specimen preparation methods (HP + LBC) and any single specimen preparation method

^*^Comparison of diagnostic positive rates among combination of the three specimen preparation methods and combination of the two specimen preparation methods; ^**&**^Comparison of diagnostic positive rates between combination of the three specimen preparation methods and any single specimen preparation method

**Table 3:** Comparison of the sensitivity, specificity, PPV, NPV and diagnostic accuracy of different specimen preparation methods for the diagnosis of lung cancer from LNs (*n* = 213)

	Sensitivity, % (95% CI)	Specificity, % (95% CI)	PPV, % (95% CI)	NPV, % (95% CI)	Diagnostic accuracy, % (95% CI)	χ^2^	*p* value
HP	89.0(83.4–93.3)	100(91.2–100)	100	67.8(57.9–76.3)	91.1(86.4–94.5)		
TSC	83.8(77.5–88.9)	95.0(83.1–99.4)	98.6(94.9–99.6)	57.6(49.0-65.7)	85.9(80.5–90.3)		
LBC	80.3(73.6–86.0)	97.5(86.8–99.9)	99.3(95.2–99.9)	53.3(45.8–60.9)	83.6(77.9–88.3)	5.51^▼^	0.064^▼^
HP + TSC	97.1(93.3–99.1)	95.0(83.1–99.4)	98.8(95.6–99.7)	86.4(76.2–94.8)	96.2(93.3–98.7)	21.15^★^	0.000^★^
HP + LBC	94.8(90.4–97.6)	97.5(86.8–99.9)	99.4(95.9–99.9)	81.2(69.6–89.1)	95.3(91.5–97.7)	18.03^■^	0.000^■^
HP + TSC + LBC	98.3(95.0-99.6)	92.5(79.6–98.4)	98.3(95.0-99.4)	92.5(80.0-97.4)	97.2(94.0–99.0)	1.04^△^, 24.69^▽^	0.595^△^, 0.000^▽^

Abbreviations; PPV = Positive predictive value, NPV = Negative predictive value

^▼^Comparison of diagnostic accuracy among HP, TSC and LBC;

^★^Comparison of diagnostic accuracy between combination of the two specimen preparation methods (HP + TSC) and any single specimen preparation method

^■^Comparison of diagnostic accuracy between combination of the two specimen preparation methods (HP + LBC) and any single specimen preparation method

^△^Comparison of diagnostic accuracy among combination of the three specimen preparation methods and combination of the two specimen preparation methods

^▽^Comparison of diagnostic accuracy between combination of the three specimen preparation methods and any single specimen preparation method

### Diagnostic consistency of different specimen preparation methods

**Fig. 2:**
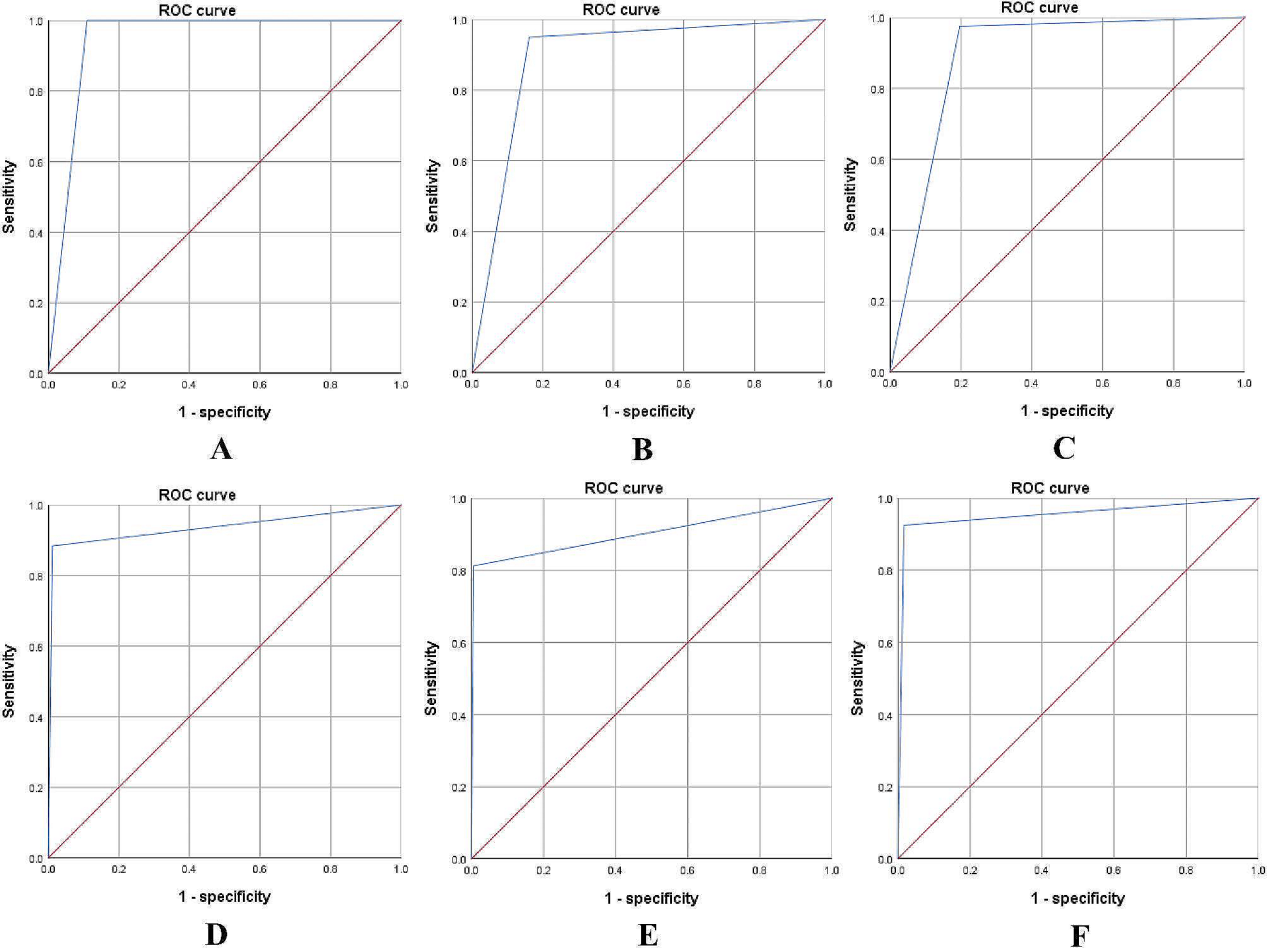
Receiver operator characteristic curve. (**A**) HP; (**B**) TSC; (**C**) LBC; (**D**) Combination of HP with TSC; (**E**) Combination of HP with LBC; (**F**) Combination of HP, LBC and TSC. (Abbreviations; HP = histopathology, TSC = traditional smear cytology, LBC = liquid-based cytology)

**Fig. 3:**
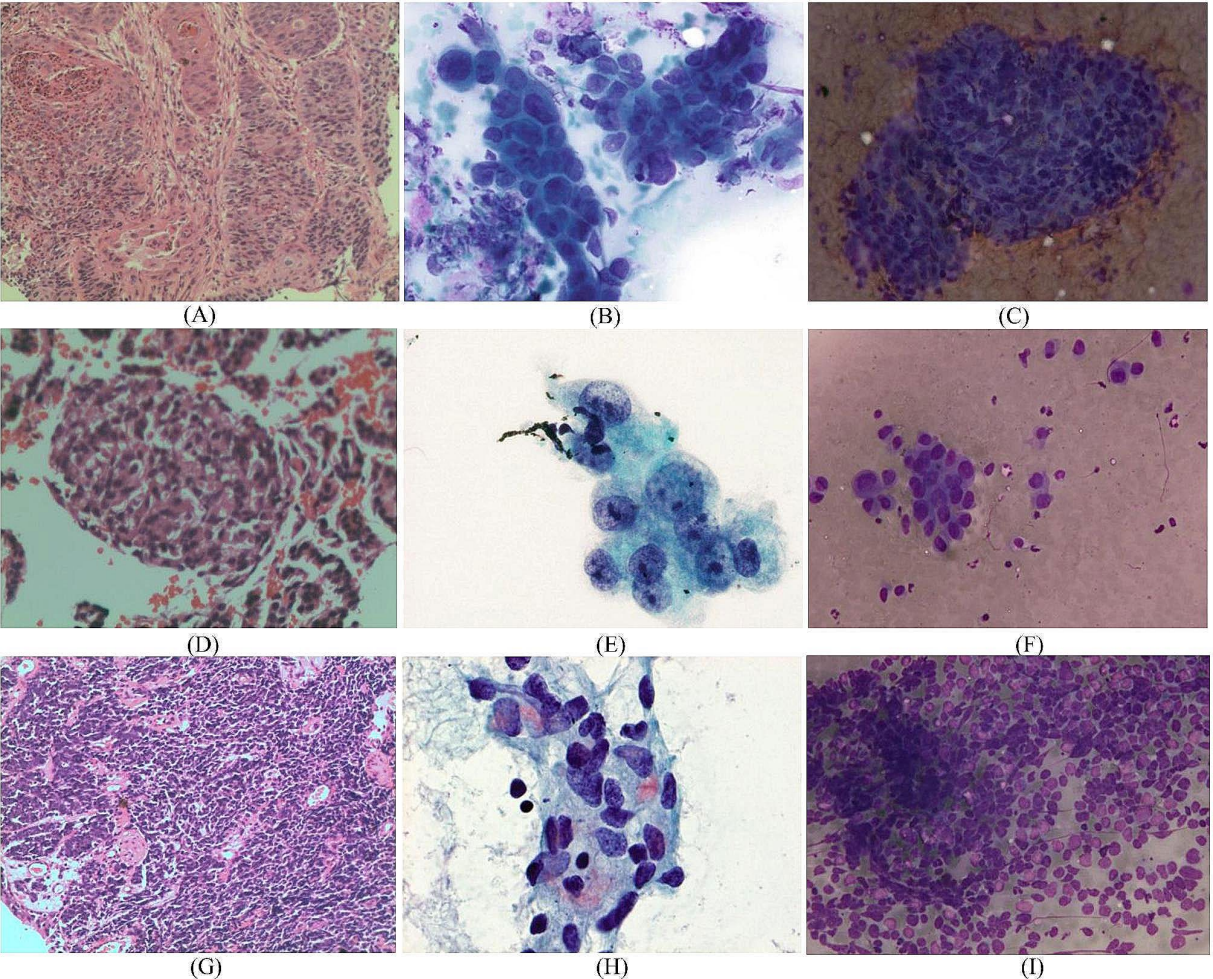
The morphological characteristics of cancer cells detected by histopathology, LBC and TSC. (**A**) Histopathological features of SqCC, (**D**) AdC, (**G**) SCC (**H**&**E**, ×400); (**B**) morphological features of LBC for SqCC, (**E**) AdC, (**H**) SCC (Pap, ×400); (**C**) morphological features of TSC for SqCC, (**F**) AdC, (**I**) SCC (**H**&**E**, ×400). (Abbreviations; SqCC = Squamous cell carcinoma, AdC = adenocarcinoma, SCC = small cell carcinoma)

As shown in Tables 4 and 5, histopathology showed a good consistency with final diagnosis (κ ± SE = 0.753 ± 0.052, *p* < 0.001), with an area under curve (AUC) of 0.945 (95% CI: 0.91–0.97, *p* < 0.001) (Fig. 2A); TSC also showed a good consistency with final diagnosis (κ ± SE = 0.631 ± 0.059, *p* < 0.001), with an AUC of 0.894 (95% CI: 0.84–0.95, *p* < 0.001) (Fig. 2B); LBC showed a general consistency with final diagnosis (κ ± SE = 0.591 ± 0.058, *p* < 0.001), with an AUC of 0.889 (95% CI: 0.84–0.93, *p* < 0.001) (Fig. 2C). As shown in Table 5, histopathology combined with TSC showed a very good consistency with final diagnosis (κ ± SE = 0.895 ± 0.039, *p* < 0.001), with an AUC of 0.936 (95% CI: 0.879–0.993, *p* < 0.001) (Fig. 2D); histopathology combined with LBC showed a very good consistency with final diagnosis (κ ± SE = 0.857 ± 0.044, *p* < 0.001), with an AUC of 0.903 (95% CI: 0.837–0.970, *p* < 0.001) (Fig. 2E); combination of the three specimen preparation methods showed a very good consistency with final diagnosis (κ ± SE = 0.908 ± 0.037, *p* < 0.001), with an AUC of 0.954 (95% CI: 0.905-1.0, *p* < 0.001) (Fig. 2F). We categorized the histological and cytological subtypes of cancer cells in accordance with the IASLC/ATS/ERS [16]. The morphological characteristics of cancer cells from different specimen preparation methods is shown in Fig. 3.

**Table 4:** Comparison of diagnostic consistency of different specimen preparation methods for the diagnosis of lung cancer from LNs (*n* = 213)

HP	Final diagnosis			TSC	Final diagnosis			LBC	Final diagnosis		
	Positive	Negative	Total		Positive	Negative	Total		Positive	Negative	Total
Positive	154	0	154	Positive	145	2	147	Positive	139	1	140
Negative	19	40	59	Negative	28	38	66	Negative	34	39	73
Total	173	40	213	Total	173	40	213	Total	173	40	213

**Table 5:** Comparison of diagnostic consistency of combination of the three or two specimen preparation methods for the diagnosis of lung cancer from LNs (*n* = 213)

HP + TSC	Final diagnosis			HP + LBC	Final diagnosis			HP + TSC + LBC	Final diagnosis		
	Positive	Negative	Total		Positive	Negative	Total		Positive	Negative	Total
Positive	168	2	170	Positive	164	1	165	Positive	170	3	173
Negative	5	38	43	Negative	9	39	48	Negative	3	37	40
Total	173	40	213	Total	173	40	213	Total	173	40	213

## Discussion

Lung cancer is one of the leading causes of cancer-related mortality around the world. To establish an appropriate treatment plan, timely diagnosis and accurate staging are essential. Today, the role of TBNA in the diagnosis and staging of lung cancer has been well established [17, 18], different studies have investigated the diagnostic accuracy of EBUS-TBNA in the diagnosis and staging of lung cancer, with always good but heterogeneous results. The accuracy of EBUS-TBNA depends not only on the endoscopist’s skills [19], but also on the specimen preparation method. There are different clinical preparation methods for TBNA specimens, such as histopathology, direct smear, liquid method, and liquid-based cytology method, etc. The specimen was placed directly onto a slide and smears were prepared on site is called direct smear technique; and specimen was deposited into a preservation bottle containing 95% alcohol and further prepared in the laboratory is called liquid method [20]. A.H. Diacon et al. [20]. conducted a prospective comparative study for TBNA material preparation and found that direct smear technique had a better positive rate than the fluid method (36.2% vs. 12.4%, respectively; *p* < 0.01). LBC is a new method of cytology preparation in recent decades. It has been widely used to screen cervical cancer and has achieved positive clinical effects. It can remove the influence of blood and mucus, make well-preserved morphological features and cleaner background [21]. In recent years, LBC is gradually applied in TBNA samples. G. Hou et al. reported a diagnostic sensitivity of 59.8% (61/102) by LBC, which was similar to that of 64.7% (65/102) by direct smear method (*p* > 0.05). And G. Gauchotte et al. [22]. concluded that the sensitivity of LBC for the diagnosis of cancer was similar to that of smear in EBUS-TBNA specimen, with no significant difference (*p* = 0.42). However, Y. Yang et al. [23]. reported that the positive rate of LBC was significant higher than that of conventional smear for bronchial lavage fluid in lung cancer patients. Of course, both TSC and LBC methods have their pros and cons, conventional smear interpretation is at times limited by the presence of air-drying artefacts, mucous, blood, and cellular overlap [3], which added difficulty in interpreting results even occurred false positives diagnosis (e.g., suspected cancer cells); the main advantages of LBC are clearer background, uniform cell thickness, and removal of air-drying artefacts [24], on the contrary, sometimes loss of background material such as necrosis and mucus also posed diagnostic dilemma in confirmation of the malignant nature of the lesion, and lead to false-negative diagnosis, as G. Gauchotte et al. concluded that LBC, if used alone, increased the risk of a false-negative result [22]. In the current study, there were several false positives in cytology, we analyzed the reasons for the false positives as follows: the preparation of TSC samples was first air-dried and then fixed with 95% alcohol. air-dried may lead to the enlargement of cell morphology or cellular overlap, which may lead to the occurrence of false positives [24]; In addition, the presence of diagnostic pitfall, for example, cases with chronic inflammatory stimulation that demonstrated reactive atypical cells were easily misinterpreted as malignancy [25, 26]. False negatives or discrepancy in the present study occurred probably due to the following reasons: malignant cells were not aspirated or not seen, or misinterpreted as benign. There were preparation artifacts which obscured cytological detail [27].

Study on the combination of conventional smear, LBC and histopathology preparation in diagnosis of lung cancer by TBNA is rare, up to now, only Y. Xu et al. [9]. have reported research data on this topic. As shown in Tables 2 and 3, compared to the single specimen preparation methods, diagnostic sensitivities and accuracies of the combination of three or two specimen preparation methods are significantly improved (*p* < 0.05), however, no significant difference was noted among combination of the three specimen preparation methods and combination of the two specimen preparation methods (histopathology and TSC, or histopathology and LBC), which is in line with the study of Y. Xu et al. [9]. . Moreover, the cost of TSC is 55 yuan (RMB) and the cost of LBC is 170 yuan (RMB) in our hospital, in this sense, considering the overall diagnostic efficiency and cost, histopathology combined with TSC is undoubtedly the best choice, this conclusion is in line with the study of Y. Xu et al. In general, the three specimen preparation methods can achieve the best results in improving the positive rate, sensitivity and accuracy, but their economic efficiency is not optimal. In some cases, LBC can be used as a complementary diagnosis to the combination of histopathology and TSC. Although TBNA procedures and pathological diagnosis were performed by experienced bronchoscopists and pathologists/cytopathologists in present study, a limitation of our study is that its retrospective, single center nature.

### Electronic supplementary material

Below is the link to the electronic supplementary material.

Supplementary Material 1

## Data Availability

No datasets were generated or analysed during the current study.

## Abbreviations

EBUS-TBNA: Endobronchial ultrasound-guided transbronchial needle aspiration
TSC: Traditional smear cytology of aspirates
LBC: liquid-based cytology of aspirates
ROC: Receiver operator characteristic
PPV: positive predictive values
NPV: negative predictive values
LNs: lymph nodes
area AUC: under curve
