# Supplementary material for: Clinical values of different specimen preparation methods for the diagnosis of lung cancer by EBUS-TBNA
Source: Diagn Pathol. 2024 Apr 19;19:61. doi: 10.1186/s13000-024-01486-1 (PMC11027543; doi:10.1186/s13000-024-01486-1)
Supplement: Supplementary file 1 — Supplementary Material 1 [file 13000_2024_1486_MOESM1_ESM.docx]

Supplemental table 1. Consistency between histopathology and final diagnosis for the diagnosis of lung cancer from LNs (n=213)

|  |  |  |  |  | Final diagnosis |  |  |  |  |  |
| --- | --- | --- | --- | --- | --- | --- | --- | --- | --- | --- |
| Histopathology | AdC | SqCC | SCLC | NSCLC | Malignant mesothelioma | lymphoma | Other malignancy | Unknown subtype | Non-malignancy | Total |
| AdC | 74 | 0 | 0 | 0 | 0 | 0 | 0 | 0 | 0 | 74 |
| SqCC | 0 | 25 | 0 | 0 | 0 | 0 | 0 | 0 | 0 | 25 |
| SCLC | 0 | 0 | 37 | 0 | 0 | 0 | 0 | 0 | 0 | 37 |
| NSCLC | 0 | 0 | 0 | 2 | 0 | 0 | 0 | 0 | 0 | 2 |
| Malignant mesothelioma | 0 | 0 | 0 | 0 | 2 | 0 | 0 | 0 | 0 | 2 |
| lymphoma | 0 | 0 | 0 | 0 | 0 | 5 | 0 | 0 | 0 | 5 |
| Other malignancy | 0 | 0 | 0 | 0 | 0 | 0 | 3 | 0 | 0 | 3 |
| Unknown subtype | 0 | 0 | 0 | 0 | 0 | 1 | 0 | 5 | 0 | 6 |
| Non-malignancy | 10 | 4 | 2 | 0 | 0 | 0 | 1 | 2 | 40 | 59 |
| Total | 84 | 29 | 39 | 2 | 2 | 6**^❈^** | 4**^**^** | 7 | 40 | 213 |

**^**^** including 1 LN of metastatic lung adeno-squamous carcinoma, 1 LN of metastatic lung sarcomatoid carcinoma, 1 LN of metastatic large cell lung carcinoma, 1 LN of metastatic clear cell renal cell carcinoma.

**^❈^** including 5 LNs of B cell non-Hodgkin's lymphoma, 1 LN of Hodgkin's lymphoma.

Supplemental table 2. Consistency between LBC and final diagnosis for the diagnosis of lung cancer from LNs (n=213)

|  |  |  |  |  | Final diagnosis |  |  |  |  |  |
| --- | --- | --- | --- | --- | --- | --- | --- | --- | --- | --- |
| LBC | AdC | SqCC | SCLC | NSCLC | Malignant mesothelioma | lymphoma | Other malignancy | Unknown subtype | Non-malignancy | Total |
| AdC | 67 | 0 | 0 | 0 | 0 | 0 | 0 | 0 | 0 | 67 |
| SqCC | 0 | 17 | 0 | 0 | 0 | 0 | 0 | 0 | 0 | 17 |
| SCLC | 0 | 0 | 29 | 0 | 0 | 0 | 0 | 0 | 0 | 29 |
| NSCLC | 3 | 1 | 1 | 2 | 0 | 0 | 2 | 3 | 0 | 12 |
| Malignant mesothelioma | 0 | 0 | 0 | 0 | 0 | 0 | 0 | 0 | 0 | 0 |
| lymphoma | 0 | 0 | 0 | 0 | 0 | 3 | 0 | 0 | 0 | 3 |
| Other malignancy | 0 | 0 | 0 | 0 | 0 | 0 | 0 | 0 | 0 | 0 |
| Unknown subtype | 4 | 0 | 3 | 0 | 1 | 0 | 1 | 2 | 1 | 12 |
| Non-malignancy | 10 | 11 | 6 | 0 | 1 | 3 | 1 | 2 | 39 | 73 |
| Total | 84 | 29 | 39 | 2 | 2 | 6**^❈^** | 4**^**^** | 7 | 40 | 213 |

**^**^** including 1 LN of metastatic lung adeno-squamous carcinoma, 1 LN of metastatic lung sarcomatoid carcinoma, 1 LN of metastatic large cell lung carcinoma, 1 LN of metastatic clear cell renal cell carcinoma.

**^❈^** including 5 LNs of B cell non-Hodgkin's lymphoma, 1 LN of Hodgkin's lymphoma.

Supplemental table 3. Consistency between TSC and final diagnosis for the diagnosis of lung cancer from LNs (n=213)

|  |  |  |  |  | Final diagnosis |  |  |  |  |  |
| --- | --- | --- | --- | --- | --- | --- | --- | --- | --- | --- |
| TSC | AdC | SqCC | SCLC | NSCLC | Malignant mesothelioma | lymphoma | Other malignancy | Unknown subtype | Non-malignancy | Total |
| AdC | 58 | 0 | 0 | 0 | 0 | 0 | 0 | 1 | 0 | 59 |
| SqCC | 0 | 17 | 0 | 0 | 0 | 0 | 0 | 0 | 0 | 17 |
| SCLC | 0 | 0 | 29 | 0 | 0 | 0 | 0 | 0 | 0 | 29 |
| NSCLC | 13 | 1 | 1 | 2 | 0 | 0 | 2 | 1 | 1 | 21 |
| Malignant mesothelioma | 0 | 0 | 0 | 0 | 0 | 0 | 0 | 0 | 0 | 0 |
| lymphoma | 0 | 0 | 0 | 0 | 0 | 2 | 0 | 0 | 0 | 2 |
| Other malignancy | 0 | 0 | 0 | 0 | 0 | 0 | 0 | 0 | 0 | 0 |
| Unknown subtype | 6 | 4 | 5 | 0 | 1 | 0 | 1 | 1 | 1 | 19 |
| Non-malignancy | 7 | 7 | 4 | 0 | 1 | 4 | 1 | 4 | 38 | 66 |
| Total | 84 | 29 | 39 | 2 | 2 | 6**^❈^** | 4**^**^** | 7 | 40 | 213 |

**^**^** including 1 LN of metastatic lung adeno-squamous carcinoma, 1 LN of metastatic lung sarcomatoid carcinoma, 1 LN of metastatic large cell lung carcinoma, 1 LN of metastatic clear cell renal cell carcinoma.

**^❈^** including 5 LNs of B cell non-Hodgkin's lymphoma, 1 LN of Hodgkin's lymphoma.
